# Robust stability of fractional order polynomials with complicated uncertainty structure

**DOI:** 10.1371/journal.pone.0180274

**Published:** 2017-06-29

**Authors:** Radek Matušů, Bilal Şenol, Libor Pekař

**Affiliations:** 1 Centre for Security, Information and Advanced Technologies (CEBIA–Tech), Faculty of Applied Informatics, Tomas Bata University in Zlín, Zlín, Czech Republic; 2 Department of Computer Engineering, Faculty of Engineering, Inonu University, Malatya, Turkey; 3 Department of Automation and Control Engineering, Faculty of Applied Informatics, Tomas Bata University in Zlín, Zlín, Czech Republic; Chongqing University, CHINA

## Abstract

The main aim of this article is to present a graphical approach to robust stability analysis for families of fractional order (quasi-)polynomials with complicated uncertainty structure. More specifically, the work emphasizes the multilinear, polynomial and general structures of uncertainty and, moreover, the retarded quasi-polynomials with parametric uncertainty are studied. Since the families with these complex uncertainty structures suffer from the lack of analytical tools, their robust stability is investigated by numerical calculation and depiction of the value sets and subsequent application of the zero exclusion condition.

## 1. Introduction

Fractional order control represents promising and attractive research topic, which has been widely studied recently. In fact, the field of fractional order calculus itself is not new [1]–[4], but the true boom of scientific works has exploded in various application areas over the last few years [5], [6]. The applications of fractional order calculus can be found, among others, in physics [7], [8], bioengineering [9]–[12], viscoelastic materials [13], [14] and also [12], chaotic systems [15]–[18], electronic circuits and fractance devices [19], [20], ultracapacitors [21], robotics [22]–[25], signal processing [26], [27], and many other areas. Certainly, the field of automatic control is no exception to this trend, quite the opposite [28]–[31]. On the other hand, the robustness of control systems can already be seen as one of the classical (and fundamental) problems in control engineering theory [32]–[36] and practice [37]. Naturally, the combination of robust and fractional order control is nowadays really appealing research discipline both for linear [38]–[61] and nonlinear [62]–[64] systems.

In control theory, a common way for the incorporation of uncertainty into mathematical model consists in the utilization of the parametric uncertainty. The systems with parametric uncertainty are supposed to have known and fixed structure (i.e. order), but some of their usually real parameters can vary (“slowly” in time) within assumed intervals. The typical problem related to the systems under parametric uncertainty is to investigate if such systems are stable (or such plants are stabilized) for all possible combinations of uncertain parameters, that is if the systems are robustly stable (or the plants are robustly stabilized). An array of methods was developed for robust stability analysis of integer order systems with parametric uncertainty [33], [34]. The selection of suitable tool depends mainly on the uncertainty structure (i.e. on relations among uncertain coefficients and complexity of used functions). Generally, the more complex uncertainty structures require more complex analysis methods. However, the value set concept combined with the zero exclusion condition [33] represents a universal graphical tool which is applicable also for the most complicated uncertainty structures [65]–[67].

As mentioned above, the robust and fractional order control has been widely combined by many researchers nowadays and thus a number of works on the issue of robust stability analysis of fractional order system have appeared lately. The robust stability test procedure for fractional order linear time-invariant (FO-LTI) systems of commensurate orders with interval uncertainty was firstly proposed in [38]. The extension to the case of systems with also interval fractional orders was discussed in [39]. Then, the robust stability problem for the general type of interval FO-LTI systems of noncommensurate orders was opened in [40]. The state-space form of the interval FO-LTI systems was considered and their robust stability tested for the first time in [41] by means of the matrix perturbation theory. The alternative approach based on the Lyapunov inequality was subsequently presented in [42]. The deficiency of the last two above-mentioned results (and also of many other works that followed) can be seen their conservativeness as the conditions are only sufficient ones. The necessary and sufficient condition for the interval FO-LTI systems was derived e.g. in [43] by using a complex Lyapunov inequality or in [44] in terms of linear matrix inequalities. However, both these works considered only the case of fractional order *α* ∈ [1, 2) and thus some further papers were focused on the *α* ∈ (0, 1) case–see e.g. [45]. Then, the robust stability of FO-LTI interval systems with linear coupling relationships among the fractional order and other model parameters were studied for the cases of *α* ∈ [1, 2) and *α* ∈ (0, 1) in [46] and [47], respectively. The robust stability and stabilization of FO-LTI systems with polytopic uncertainty was considered e.g. in [48]. However, the systems with more complicated uncertainty structures suffer from the lack of, especially analytical, tools. An exceptionally universal method is represented by the combination of the value set concept and the zero exclusion condition. Its classical integer order version [33] was extended to the fractional order cases e.g. in [49]–[53].

This article presents a graphical approach to the robust stability analysis of families of fractional order polynomials (which can be considered as characteristic polynomials of investigated fractional order systems) with a particular emphasis on families of polynomials with complicated uncertainty structure based on plotting the numerically obtained value sets and utilization of the zero exclusion condition. This work is intended to accompany the contribution [51] and to put a stress on complex uncertainty structures such as multilinear, polynomial, and general, or even on the uncertain quasi-polynomials arising from the application of time-delay models.

The article is organized as follows. In Section 2, the fractional order polynomials with parametric uncertainty are defined. The Section 3 describes various structures of uncertainty and outlines the typical tools for their robust stability analysis. The graphical approach to robust stability investigation based on the value set concept and the zero exclusion condition is presented in Section 4. Further, Section 5 shows the practical applicability of the method by means of four illustrative examples with various complicated uncertainty structures. And finally, Section 6 offers some conclusion remarks.

## 2. Fractional order polynomials with parametric uncertainty

General continuous integro-differential operator (differintegral) is defined as [5], [28], [30]:

Datα={dαdtαRe α>01Re α=0∫at(dτ)−αRe α<0 (1)

where *α* is the order of the differintegration (typically *α* ∈ ℝ) and *a* and *t* are the limits of the operation. The differintegral can be defined in various ways. The three most common are Riemann-Liouville, Grünwald-Letnikov and Caputo definitions.

The Laplace transform of the differintegral which is defined in the Riemann-Liouville way is given by [3], [28], [30]:

L{D0tαf(t)}=∫0∞e−stD0tαf(t)dt=sαF(s)−∑m=0n−1smD0tα−m−1f(t)|t=0 (2)

where integer *n* lies within (*n*– 1 < *α* ≤ *n*). Under the assumption of zero initial conditions, the Laplace transform is simply [30]:

L{D0tαf(t)}=sαF(s) (3)

which holds true for all three mentioned differintegral definitions.

The fractional order polynomial with parametric uncertainty has the form:

$$p(s,q)=\rho_{n}(q)s^{\alpha_{n}}+\rho_{n-1}(q)s^{\alpha_{n-1}}+\cdots +\rho_{1}(q)s^{\alpha_{1}}+\rho_{0}(q)s^{\alpha_{0}}$$ (4)

where *q* is the vector of uncertainty, *α*^*n*^ > *α*^*n*−1^ > ⋯ > *α*^1^ > *α*^0^ are real numbers and *ρ*_*i*_ for *i* = 0,…,*n* are coefficient functions.

The family of fractional order polynomials is then [33]:

P={p(⋅,q):q∈Q} (5)

where *Q* is the uncertainty bounding set (commonly considered as a multidimensional box, i.e. individual components of vector *q* are bounded by intervals).

## 3. Structures of uncertainty

A level of complicatedness of the relations among coefficients of the polynomial Eq (4) (in other words the complexity of the coefficient functions *ρ*_*i*_ and their interconnections) is a crucial factor for the decision on a suitable tool for robust stability analysis both for integer and fractional order systems with parametric uncertainty. According to this, one can distinguish among several kinds of uncertainty structures. Standard classification for integer order systems is [33], [65], [66]:

- Independent uncertainty structure (called interval one for *Q* in the shape of a box)
- Affine linear uncertainty structure (called polytopic one for *Q* in the shape of a polytope)
- Multilinear uncertainty structure
- Polynomial (polynomic) uncertainty structure
- General uncertainty structure

On top of that, so-called single parameter uncertainty is a special case, which can be seen as the simplest one despite the structure itself can be formally affine linear or even more complicated.

In the interval uncertainty, each uncertain parameter may enter into the one and only coefficient (although theoretically more uncertain parameters can enter into the same coefficient). This results in the mutual independence of all coefficients and possible utilization of the famous Kharitonov theorem. However, this holds true only for integer order version of interval polynomials. The case of fractional order interval polynomials is a bit more complicated since the real and imaginary parts can be mutually dependent and thus, the classical Kharitonov theorem is not directly applicable anymore (see e.g. [49]).

The affine linear uncertainty structure means that more uncertain parameters can enter into the same coefficient, but these coefficients must have the form of affine linear functions, i.e.:

$$\rho_{i}(q)=\left(a_{k}q_{k}+a_{k-1}q_{k-1}+\cdots +a_{1}q_{1}+a_{0}\right)$$ (6)

where *k* is the dimension of the uncertainty vector *q* and *a*_*m*_ are constants for *m* = 0,…,*k*. The affine linear uncertainty structure appears very commonly in robust control practice because a simple interval controlled plant in feedback connection with a fixed controller generally leads to a closed-loop characteristic polynomial with affine linear uncertainty structure. A number of tools for investigation or robust stability for this structure can be found in the integer order robust control literature (e.g. the edge theorem, the 32 edge theorem (or similar generalized Kharitonov theorem) and more specialized 16 plant theorem). Robust stability of fractional order systems with affine linear uncertainty structure has been studied e.g. in [48].

The next and more complex level of relations among polynomial coefficients is represented by the multilinear uncertainty structure. It means that if all but one uncertain parameters are fixed, then *ρ*_*i*_ is affine linear in the remaining (non-fixed) parameter. Practically speaking, the coefficients can contain the product of uncertain parameters. The robust stability analysis for this uncertainty structure can already be quite a complicated task because the value sets are non-convex and tools based on extreme points or edges are not valid anymore. Well-known result for integer order polynomials with multilinear uncertainty structure is the mapping theorem, which works with the convex hull of the original family. Consequently, the analysis is easier but the cost is the sufficiency of the obtained results. A possible technique for investigation of fractional order polynomials with multilinear uncertainty structure can be found e.g. in [52].

The family with polynomial (polynomic) uncertainty structure contains the coefficient functions *ρ*_*i*_ with multivariable polynomials in uncertain parameters. The robust stability analysis is even more complicated because the value sets are not only non-convex but moreover, they can protrude from the convex hulls of the extremes. The polynomial uncertainty structure can be formally transformed into the multilinear one with different uncertainty bounding set but it is not very useful from the analysis point of view.

Finally, in general uncertainty structures the coefficients *ρ*_*i*_ can be arbitrary multivariable functions of components of *q* provided that *ρ*_*i*_ are continuous functions on assumed intervals. Practically no analytical tools are available for robust stability investigation in this general case.

Besides all the mentioned uncertainty structures the special type of retarded quasi-polynomial is a frequent object of interest from the control theory viewpoint. Assume (integer or fractional order) controlled time-delay plant:

$$G(s,q,\Theta )=\frac{b(s,q)}{a(s,q)}e^{-\Theta (q)s}$$ (7)

where not only numerator *b*(*s*,*q*) and denominator *a*(*s*,*q*) polynomials are uncertain, but also the time-delay term Θ(*q*) can vary within supposed bounds (formally it could be included in the same vector of uncertainty *q*). Then suppose the plant Eq (7) is in the classical feedback connection with (integer or fractional order) controller:

$$C(s)=\frac{c_{N}(s)}{c_{D}(s)}$$ (8)

The corresponding uncertain (integer or fractional order) closed-loop characteristic retarded quasi-polynomial is:

$$p_{CL}(s,q,\Theta )=a(s,q)c_{D}(s)+b(s,q)e^{-\Theta s}c_{N}(s)$$ (9)

Simple graphical analysis of robust stability for this kind of fractional order quasi-polynomial is shown in [58].

## 4. Value sets and zero exclusion condition

As mentioned above, the complicated structures of uncertainty suffer from the lack of suitable techniques for robust stability analysis. However, a graphical method based on the combination of the value set concept and the zero exclusion condition [33] represents a universal tool, which can be applied to a wide range of uncertainty structures, including the most complex ones. Besides this, it can be used also for various regions of stability (robust *D*-stability). More details on parametric uncertainty, related robust stability analysis and several examples of the typical value sets for the integer order systems can be found in [33] and subsequently e.g. in [65], [66]. The works [49]–[53] extended the concept of the value set to fractional order uncertain polynomials (or quasi-polynomials [58]).

The value set for the family of polynomials Eq (5) at the frequency *ω* ∈ ℝ is defined as [33]:

p(jω,Q)={p(jω,q):q∈Q} (10)

which means that *p*(*jω*, *Q*) is the image of *Q* under *p*(*jω*,·). In practice, the value sets can be constructed by substituting *s* for *jω*, fixing *ω* and letting the vector of uncertain parameters *q* range over the set *Q*.

The zero exclusion condition for (Hurwitz) stability of the family of continuous-time polynomials Eq (5) can be formulated [33]: Consider the invariant degree of polynomials in the family, pathwise connected uncertainty bounding set *Q*, continuous coefficient functions *ρ*_*k*_(*q*) for *k* = 0, 1, 2,…, *n* and at least one stable member *p*(*s*, *q*^0^). Then the family *P* is robustly stable if and only if the origin of the complex plane (zero point) is excluded from the value set *p*(*jω*, *Q*) at all frequencies *ω* ≥ 0, i.e. *P* is robustly stable if and only if:

0∉p(jω,Q) ∀ω≥0 (11)

Note that this universal approach is directly applicable also for the families of retarded quasi-polynomials with the structure Eq (9) [33], [58], [65], [68].

**Fig 1:**
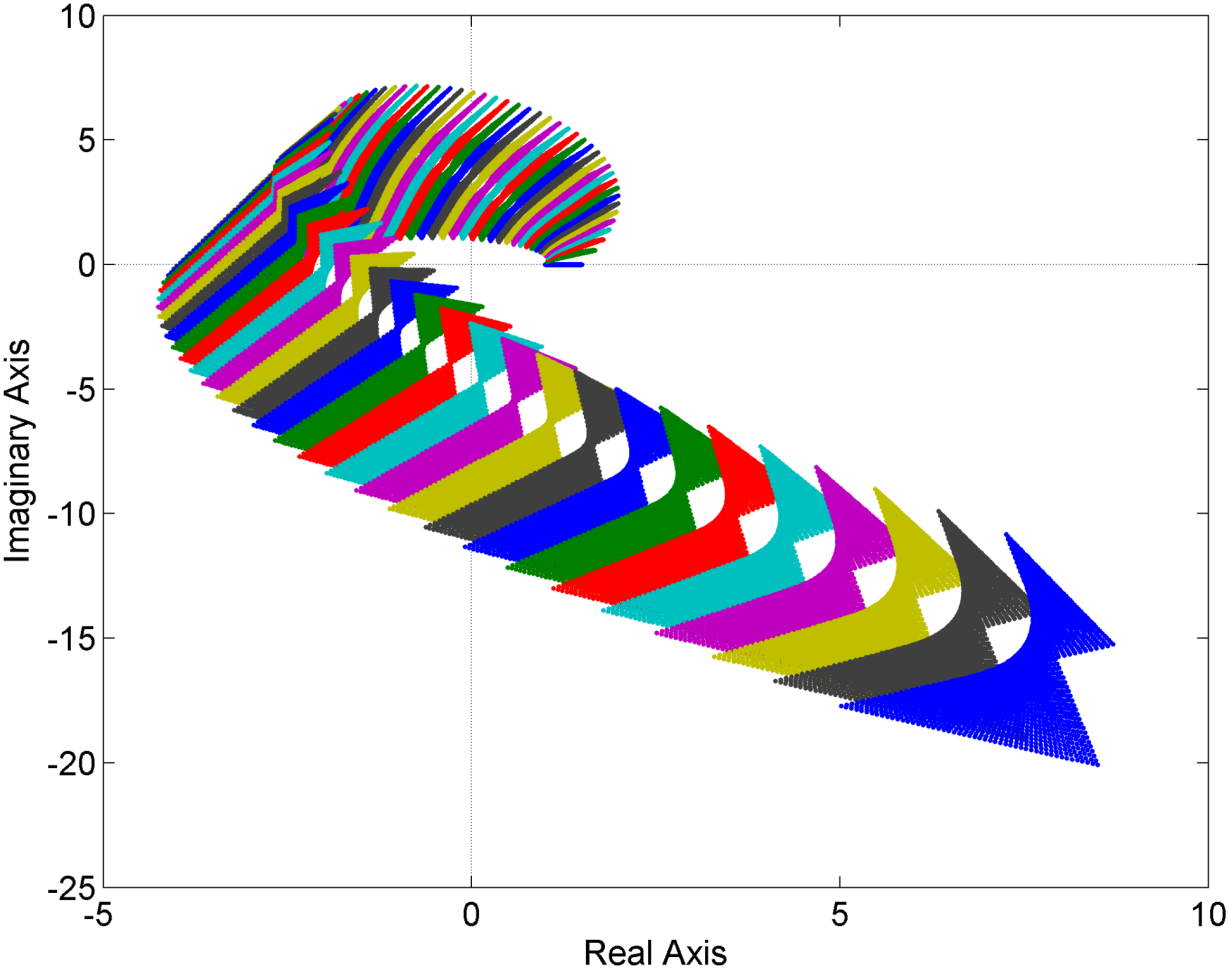
Value sets of the family of fractional order polynomials with multilinear uncertainty structure Eq (12).

**Fig 2:**
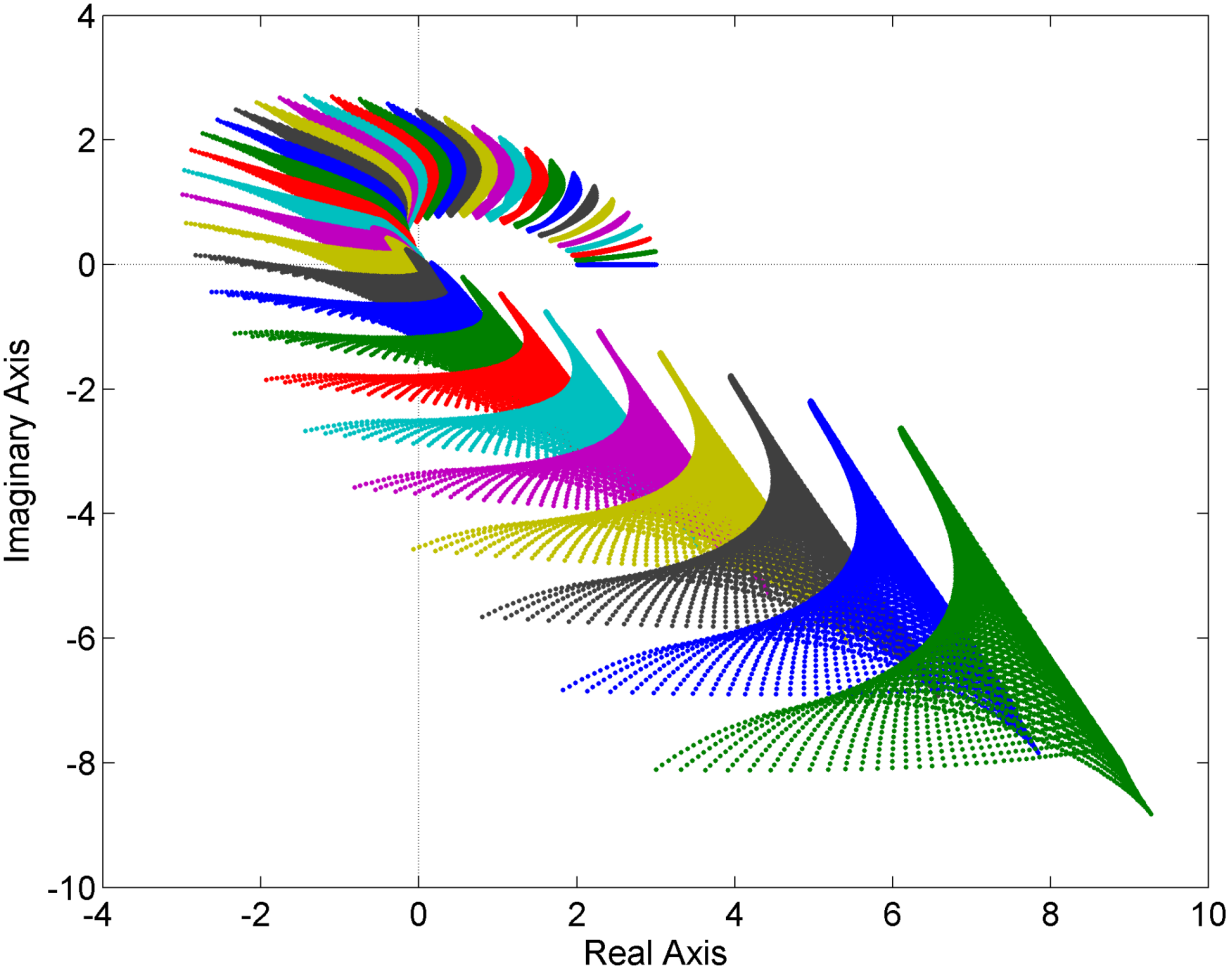
Value sets of the family of fractional order polynomials with polynomial uncertainty structure Eq (13).

**Fig 3:**
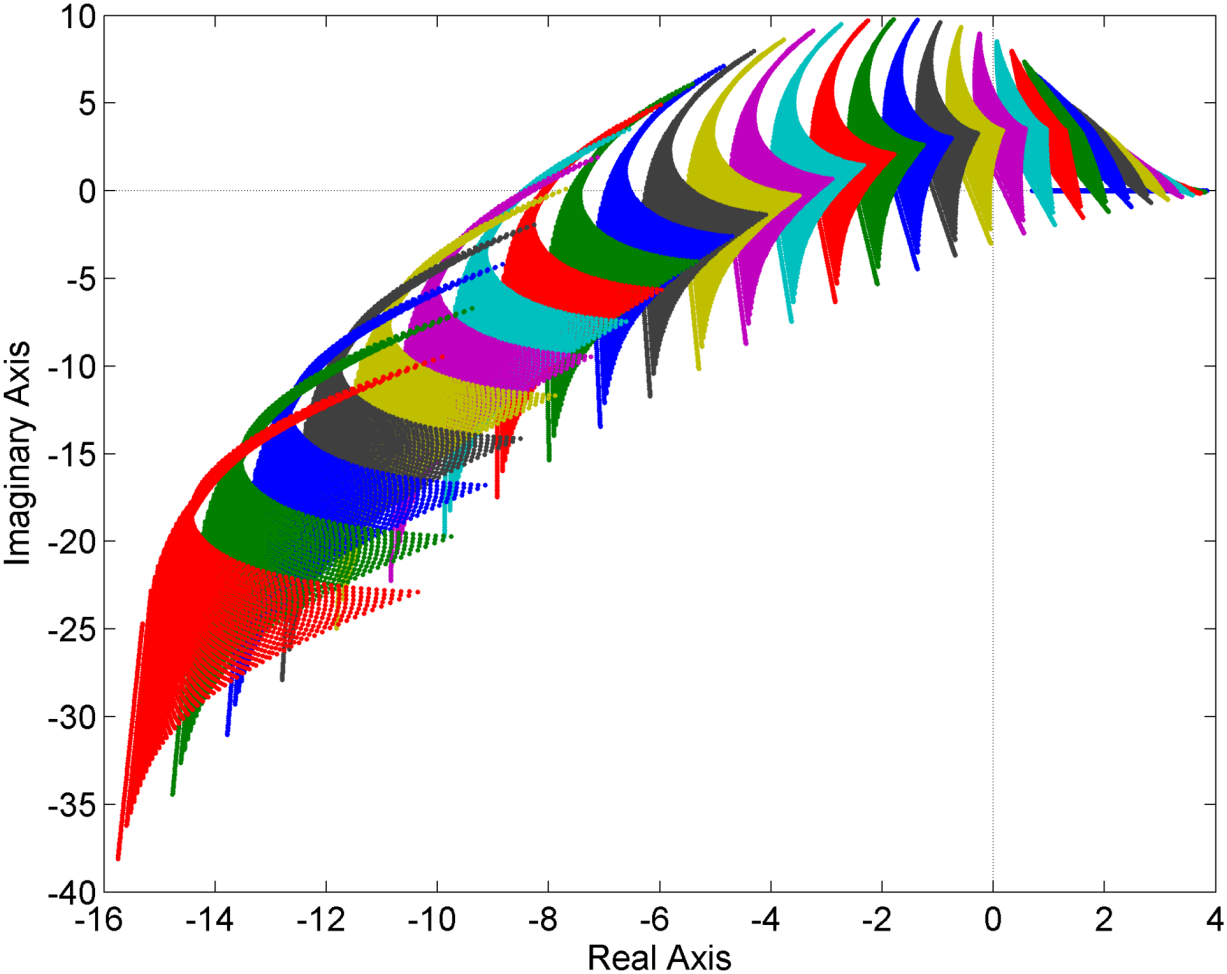
Value sets of the family of fractional order polynomials with general uncertainty structure Eq (14).

**Fig 4:**
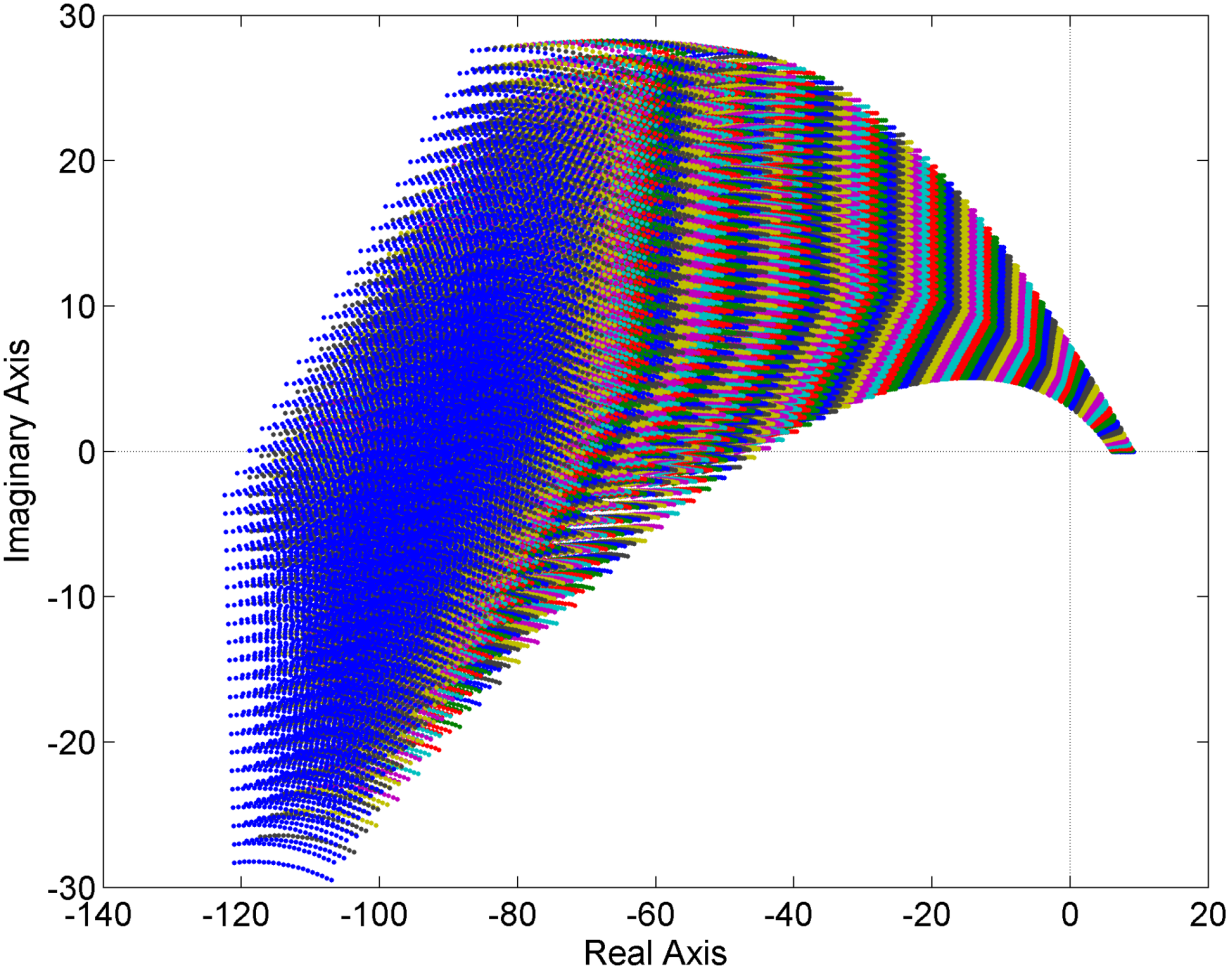
Value sets of the family of fractional order retarded quasi-polynomials Eq (18).

In this work, the value sets for the fractional order families with complicated uncertainty structures are plotted by using a suitable sampling (gridding) of the uncertain parameters and direct calculation of related partial points of the value sets for a supposed frequency range. It means that the practical plotting of the Figs 1–4 from the next Section 5 and evaluation of the robust stability tests were performed as follows. A suitable set of non-negative frequencies was pre-selected and then the value set for each individual frequency was depicted. All those individual value sets are composed of the points corresponding to the images of all variations of the appropriately sampled uncertain parameters. When the suitable value sets were obtained, their position in relation to the origin of the complex plane had to be checked. As defined above, the family of (quasi-)polynomials is robustly stable if and only if the complex plane origin (zero point) is excluded from the value sets and all other required assumptions are fulfilled, especially the existence of at least one stable member of the family. This existence could be actually verified before the graphical analysis and if a chosen member is found unstable, the graphical test itself can be skipped because the whole family is robustly unstable. This technique is relatively easy-to-use, it leads to the robust stability results with the necessary and sufficient condition, and it is applicable even for the systems with very complicated uncertainty structures, which represents its main advantage. On the other hand, a long computational time for a high number of uncertain parameters is the weakness.

## 5. Illustrative examples

In order to show the practical applicability of the graphical approach to robust stability analysis discussed hereinbefore, four illustrative examples with families of fractional order (quasi-)polynomials are presented in this Section. The first three examples deal with multilinear, polynomial and general uncertainty structure, successively, and the last one focuses on a family of retarded quasi-polynomials.

### 5.1 Multilinear uncertainty structure

First, assume the family of fractional order polynomials with multilinear uncertainty structure:

$$p_{MUL}(s,q)=s^{4.1}+\left(q_{1}q_{2}+3\right)s^{3.2}+\left(q_{1}+q_{2}+6\right)s^{1.9}+\left(2q_{1}q_{2}+5q_{1}+q_{2}+2\right)s^{0.8}+0.5q_{1}q_{2}+1$$ (12)

where both *q*_1_ ∈ [0,1] and *q*_2_ ∈ [0,1].

The value sets depicted for the frequency range from 0 to 2.1 [rad/s] with the step 0.03 by means of sampling the uncertain parameters (with the step 0.02) are shown in Fig 1. In accordance with the process described in the previous Section, the family Eq (12) is robustly stable because it contains a stable member and the origin of the complex plane (zero point) is excluded from the plotted value sets.

### 5.2 Polynomial uncertainty structure

In the second example, consider the family of fractional order polynomials with polynomial (polynomic) uncertainty structure:

$$p_{POL}(s,q)=s^{4.2}+\left(q_{1}^{3}+q_{2}^{3}+2\right)s^{3.3}+\left(3q_{1}^{5}q_{2}^{2}+q_{1}q_{2}+5\right)s^{1.8}+\left(q_{1}^{2}q_{2}+q_{1}q_{2}^{2}+1\right)s^{0.9}+q_{1}q_{2}+2$$ (13)

where again *q*_1_ ∈ [0,1], *q*_2_ ∈ [0,1].

The value sets are now plotted for the frequency range 0:0.05:1.8 [rad/s] and both uncertain parameters are sampled with the step 0.01 –see Fig 2. Obviously, the family Eq (13) is not robustly stable because the complex plane origin is included in the value sets.

### 5.3 General uncertainty structure

The third example deals with the family of fractional order polynomials with completely general uncertainty structure:

$$\begin{array}{l} p_{GEN}(s,q)=s^{3.2}+\left(\text{cos}\left(q_{1}q_{2}\right)+2\right)s^{2.1}+\left(5\sqrt{\left|q_{1}\right|}-3\text{sin}\left(q_{2}\right)-\text{cos}\left(q_{1}q_{2}\right)+3\right)s^{0.9}+\cdots \\ +\left(-\sqrt{\left|q_{1}\right|}+\text{sin}\left(q_{2}\right)+\text{cos}\left(q_{1}q_{2}\right)+2\right) \end{array}$$ (14)

where *q*_1_ ∈ [−1,1], *q*_2_ ∈ [−1,1].

The use of the frequency range 0:0.1:3 [rad/s] and sampling the uncertain parameters with the step 0.02 lead to the value sets which are shown in Fig 3. Similarly as in the previous case, the family Eq (14) is robustly unstable due to the inclusion of the zero point in the value sets.

### 5.4 Uncertain quasi-polynomials

The aim of the last example is to decide on the robust (in)stability of the family of retarded quasi-polynomials. In [69], the fractional order PI controller:

$$C(s)=4.0494+\frac{7.6646}{s^{1.45}}=\frac{4.0494s^{1.45}+7.6646}{s^{1.45}}$$ (15)

was designed for the first order plus time delay plant:

$$G_{0}(s)=\frac{K_{0}}{T_{0}s+1}e^{-\Theta_{0}s}=\frac{1}{s+1}e^{-0.1s}$$ (16)

For the purpose of this article, a potential change of ±20% in the gain, time constant, and time-delay term is supposed, i.e.:

$$G(s,K,T,\Theta )=\frac{K}{Ts+1}e^{-\Theta s}=\frac{\left[0.8,1.2\right]}{\left[0.8,1.2\right]s+1}e^{-\left[0.08,0.12\right]s}$$ (17)

According to Eq (9), the corresponding family of fractional order closed-loop characteristic retarded quasi-polynomials is:

$$p_{CL}(s,K,T,\Theta )=\left(Ts+1\right)s^{1.45}+Ke^{-\Theta s}\left(4.0494s^{1.45}+7.6646\right)$$ (18)

where *K* ∈ [0.8,1.2], *T* ∈ [0.8,1.2] and Θ ∈ [0.08,0.12].

The value sets are depicted for the frequency range *ω* = 0:0.1:7 [rad/s] and for the sampled gain *K* = 0.8:0.02:1.2, time constant *T* = 0.8:0.02:1.2, and time delay term Θ = 0.08:0.002:0.12. The result can be seen in Fig 4 which clearly demonstrates that the family Eq (18) is robustly stable (the zero point is excluded and the family contains a stable member).

## 6. Conclusion

This article was focused on a graphical approach to robust stability investigation for families of fractional order polynomials or even quasi-polynomials with complicated uncertainty structure. The four illustrative examples demonstrated the application of the values set concept and the zero exclusion condition for the families of fractional order polynomials with multilinear uncertainty structure, polynomial uncertainty structure, general uncertainty structure, and for the family of the fractional order retarded quasi-polynomials. The obtained results showed the effectivity of the method for robust stability analysis of fractional order polynomials with various complex uncertainty structures. The potential directions for future research can be seen in robust stability analysis of e.g. fractional order anisochronic systems with internal delays and uncertain parameters [67], fractional order systems with spherical uncertainty [70] or fractional order systems with complicated uncertainty structures combined with the uncertain fractional orders.
